# A Manual of Physiology: A Text-Book for Students

**Published:** 1884-12

**Authors:** 


A Manual of Physiology: A Text-Book for Students. *By* Gerald F. Yeo, M.D. F.R.C.S. *Professor of Physiology in King's College, London. Octavo, pp. xxiv, 749 Philadelphia:* P. Blakiston, Son & Co. 1884.

Michæl Foster's text-book on physiology will meet no mean rival in the book before us. The style is vigorous, and the author expresses himself with an enviable degree of clear_

ness and distinctness. It not as complete a book as Foster's, but at the same time, better calculated to fulfill the part of an elementary treatise. Without the omission of important facts, great care has been taken to exclude doubtful and erroneous doctrines. The woodcuts are good and add materially to the value of the book. The chapter on bibliography is wanting. We commend Yeo's physiology to the medical student and practitioner alike.

